# Employment challenges for persons with visual impairment in Windhoek, Namibia

**DOI:** 10.4102/ajod.v13i0.1500

**Published:** 2024-10-30

**Authors:** Kelao Uiras, Nisha A. Paulse, Annelisa Murangi, Clifford K. Hlatywayo

**Affiliations:** 1 Department of Psychology and Social Work, Faculty of Health Sciences and Veterinary Medicine, University of Namibia, Windhoek, Namibia; 2 Optentia Research Unit, Faculty of Humanities, North-West University, Vanderbiljpark, South Africa

**Keywords:** seeking employment, maintaining employment, persons with visual impairment, Windhoek, Namibia

## Abstract

**Background:**

The greatest challenge for persons with disabilities is that of securing and maintaining employment, because of the limitations associated with being visually impaired.

**Objectives:**

This study aims to explore the employment challenges of securing and maintaining employment faced by persons with visual impairments.

**Method:**

A qualitative research approach with an exploratory research design was employed. A non-probability sampling method using the snowball sampling technique was adopted. A semi-structured interview was conducted with a total of *n* = 9 participants, and the data were analysed using the thematic analysis method.

**Results:**

The findings indicate that persons with visual impairment continue to face adverse challenges, especially in the quest of securing employment. Discrimination, isolation, rejection and lack of recognition are key issues that continue to persist in their life experiences, from the tertiary to post-tertiary level.

**Conclusion:**

Persons with visual impairment need continued support from various stakeholders as far as employment opportunities are concerned. There is a need to sensitise stakeholders, at all levels, on the challenges encountered by persons with visual impairment in their employment journey for effective inclusion and diversity management.

**Contribution:**

The findings can aid in the development of an all-round model of support and optimal functioning for persons with visual impairment from grassroots level to post-tertiary education and in workplaces.

## Introduction

Article 27 of the UN Convention on the Rights of Persons with Disabilities (UNCRPD) (UN Convention 2006) establishes that state parties should recognise the right of persons with disabilities to work on an equal basis with others. Similarly, the Namibia Government Gazette (1998) advocates strongly for an equitable representation of persons with disabilities in the workplace. However, despite these efforts, the employment for persons with disabilities in Namibia remains a challenge (Namibia Statistics Agency 2016). This could be because most employers are reluctant to employ persons with visual impairment because of the reasonable accommodation expectations that come with it (Brunes & Heir 2022). The goal of reasonable accommodation in the workplace is to allow persons with disabilities to perform their official tasks while also enjoying a non-discriminatory and inclusive environment (UNCRPD 2006).

For persons with visual impairment, the challenge not only lies in successfully completing tertiary education, but also in securing and maintaining employment. As a result of limitations associated with each disability, there is a notable disparity in the employment rates between individuals with impairments and those without. According to the World Report on Disability (2011), persons with disability have a disproportionately lower employment rate (44%) compared to those persons without a disability (75%). Severity of visual disability (with imposed functional limitations), and levels of education also contributed significantly to whether persons with visual impairments secured employment or not (Ebuenyi et al. 2020; Lund & Cmar 2019). It can be argued that higher levels of education imply that an individual with visual impairment can read braille, and as such can be accommodated in workplaces where such reasonable adjustments are prioritised.

The ability to be employable and engage in paid work is important for many individuals, including those with disabilities (Moerdyk et al. 2020). Unemployment for persons with disabilities leads to increased poverty rates as well as increased dependency and less legal protection compared to persons without disabilities (Ikela 2019). It is evident in disability-related literature that profitable employment possibilities are necessary for self-fulfilment, personal autonomy, identity and status (Antonelli, O’Mally & Steverson 2018a; Capella McDonnall, Zhou & Grudden 2013; Nagle 2001). Having paid employment allows persons with visual disabilities to experience a higher quality of life, with a high self-reported life satisfaction and reduced levels of depression (Brunes & Heir 2022).

A lack of income or none, may also be indicated by the difficulty to find employment in the labour market, which further erodes one’s ability to meet fundamental basic needs, such as clothes, food, housing and access to postsecondary education services. As such, employment is important for people with disabilities as it not only provides financial assistance but may also provide access to better health care, improve living arrangements, promote self-reliance and reduce dependency (Antonelli, Steverson & O’Mally 2018b).

The term, *disability*, according to the UN Convention on the Rights of Persons with Disabilities (UNCRPD 2006):

[*I*]ncludes those who have long-term physical, mental, intellectual or sensory impairments which in interaction with various barriers may hinder their full and effective participation in society on an equal basis with others. (p. 4)

In the context of the current study, the word visual impairment refers to the partial or total loss of vision (Brunes & Heir 2022).

It is necessary to narrate the employment challenges of persons with visual impairment, as distinctively unique from other forms of disabilities as defined by the UNCRPD. This study, being fairly novel in Namibia, aims to explore the challenges persons with visual disabilities face in securing and maintaining employment.

## Literature review

In conceptualising disability, this study draws from the UNCRPD (2006) as a guiding framework that emphasises that:

[*P*]ersons with disabilities include those who have long-term physical, mental, intellectual or sensory impairments which in interaction with various barriers may hinder their full and effective participation in society on an equal basis with others. (p. 4)

Visual impairment, a category of sensory impairment, is defined as a condition of reduced visual functioning, characterised by partial or complete vision loss, thereby diminishing an individual’s ability to engage in activities that require the use of vision such as writing and reading (Naipal & Rampersad 2018).

## Employment experiences for persons with visual impairment

The ability to work should be seen as fundamental for fulfilment and optimal functioning of individuals (Van der Klink et al. 2016). As far as employment is concerned, Duquette and Baril (2013) mention that visually impaired people have always been underrepresented in the workplace. The stigma against disabilities thus had a large impact on their ability to secure and maintain a job. According to a study by Portillo-Navarro et al. (2022), employers do not always understand the abilities or the competencies of people with disabilities. Strindlund, Abrandt-Dahlgren and Ståhl (2019) argue that some employers view the concept of disability as a constraint to work performance and that because of the actual disabilities that people had, employers deemed them as less competent and time, energy and resource-demanding (Strindlund et al. 2019). This suggests that the negative beliefs and prejudices continue to create barriers to employment, because the misconceptions from employers added to the ignorance about available adjustments to work arrangements to accommodate employees with disabilities. Antonelli et al. (2018b) further report that negative employer attitude is one major factor influencing individuals’ ability to secure employment.

Chichaya, Joubert and McColl (2020) contend that individuals with disabilities struggled with employment and earned less than their counterparts who did not have any disabilities, which could be argued to impact their overall well-being. According to Vanhercke et al. (2014), on the overview on the relationship between perceived employability and well-being, an individual that believed that he and/or she could obtain and maintain employment was more likely to have a better wellbeing than those that did not. It was further reported that individuals who are visually impaired and have a job are less likely to suffer from symptoms of anxiety and low self-esteem (McDonnall & Antonelli 2018). In addition, a study by Guerette and Smedena (2011) pointed out that when quality of life was measured objectively, people with disabilities had a lower quality of life relative to individuals without disabilities. According to the World Disability Report, ‘people with disabilities are more likely to be unemployed and generally earn less even when employed’ yet their personal expenses may have been more than their peers without disability (World Disability Report 2011:11).

## Theoretical framework

The Social Model of Disability has been used to anchor the experiences of individuals with visual impairments in the present study. According to this model, disability is a socially constructed phenomenon that may be addressed via radical social transformation (Retief & Letsosa 2018).

In conceptualising disability, the social model argues that any form of disability is regarded as a situation caused by social conditions (such as structures and systems), which requires for its elimination, that persons with disabilities, must be capacitated by the human created structures and systems, to assume control over their own lives and live fulfilling lives (Retief & Letsosa 2018). To enable equal participation of persons with disabilities in social and economic reforms as advocated for by the various disability policies and frameworks, there is need for physical, social, communication and economic barriers to change (UNCRPD 2006).

The theory accurately interprets the experiences of marginalised groups when it comes to employment in Namibia. The theory is crucial as it is based on the unwarranted isolation and exclusion from complete involvement in society (Finkelstein 2001; Terzi 2004). Barnes (1998) advocates for the social model of disability, arguing that without it, disability studies would be stripped of meaning. This study adopts the theory as proposed by Lang (2007) who examined the social model of disablement, in arguing that the social model is not seen as a single entity, but rather a collection of different methods to comprehending the concept of disablement. Considering the principles of organisational justice, inclusivity and compliance with Namibia’s equity laws, it is critical for the subject matter to be objectified from the lens of persons with disabilities. In adopting the theory, one may contend that the experiences of persons with disabilities serve as a reflection of the precise areas in which society is erring (Finkelstein 2001). It is critical to note that the terms employability and employment are defined only from the lens of the able bodies. To enhance meaningfulness, the social model of disability is thus applicable because of its focus on one’s economic or social disadvantaged position, because of social setting and personal traits (Samaha 2007). Specific to the Namibian context, the social model of disability affords persons with visual impairment an opportunity to narrate their employment experiences in a meaningful way.

## The current study

At a global level, persons with disabilities continue to face challenges in securing and maintaining employment (Smith 2002). According to Garcia et al. (2016), the inclusion of people with disabilities is necessary for their social integration and financial independence and is argued to contribute to their well-being. The challenge, irrespective of tertiary education opportunities made available, is to secure employment and to also maintain it. Research by Chichaya et al. (2020) indicates that people with visual impairment who have acquired a job are less likely to experience anxiety and self-esteem issues.

Employees (including those with visual impairment) are recruited on the basis of their knowledge and skills (Smith 2002). Specific to Namibia, the Government of Namibia introduced the concept of special education and inclusive education to capacitate persons with disabilities with knowledge and skills to participate on an equal basis with others in the quest of securing and maintaining employment. A university degree significantly increases one’s chances of finding work (Eide et al. 2011; Namibia Planning Commission [NPC] 2018).

Despite these educational developments, to date, the struggle of employment for persons with disabilities continues to persist (Namibia Statistics Agency 2016). It is not clear what the challenges are, because of the lack of empirical findings on the employment challenges faced by those with visual impairments.

Disability-related empirical research in Namibia focused on cultural beliefs regarding people with disabilities in Namibia (Haihambo & Lightfoot 2010), implementation of the Disability Framework in Namibia (Shumba & Moodley 2018) and living conditions among people with disabilities in developing countries (Eide et al. 2011). There is a lack of qualitative empirical studies on the employment challenges faced by persons with visual impairment, necessitating the current study.

## Research methods and design

The study utilised an exploratory qualitative research design. Semi-structured interviews were conducted with n=9 persons with visual disabilities. The focus of this study was only on persons with visual disabilities who had acquired some form of educational qualifications, which is the basis for competing in the labour market for work opportunities. The participants were only recruited from Windhoek, in the Khomas Region, Namibia. A non-probability sampling method, using the snowball sampling technique, was used. Owing to the relatively small population of persons with visual impairment, snowball sampling was utilised as participants were able to refer researchers to other participants who are visually impaired.

### Research study setting and population

The study population comprised persons with visual impairment based in the capital city, Windhoek, at the time of the study. The exact number of persons with visual impairment could not be obtained. There were no restrictions on how long they have worked or have been searching for employment. The focus was on getting participants who had the experience of searching and finding employment as well as on participants who are still searching for employment after having acquired some sort of educational qualification. According to the Namibia Disability Report, most persons with disabilities are in Windhoek, where employment opportunities can be found (Namibia Statistics Agency 2016).

### Data-collection process

A semi-structured interview guide was developed for the purposes of this study. The literature on challenges faced by persons with visual impairment in securing and maintaining employment as well as the theoretical framework, guided the formulation of the interview questions.

Example of interview questions included: how would you describe your experience with searching for a job?; elaborate on the challenges faced in trying to acquire employment; describe how your type of disability may have possibly directly or indirectly impacted your experience in searching for a job.

Interviews were conducted face-to-face at an agreed upon time and in a secure environment. The researchers approached the Namibia Federation of the Visually Impaired in Namibia, and contact numbers of possible participants were provided. The researchers called the participants to narrate the purpose of the study and their possible involvement. Some were not willing to participate, and some contact numbers went unanswered. The few that the researchers could get a hold of, were able to refer to their friends, who were also visually impaired. In all data-collection attempts, researchers went to where the participants were. Before interviews commenced, researchers informed participants (face to face), on the purpose of the study and that they could opt out of the study at any given point without incurring any negative consequences. Data were tape-recorded, and in cases where participants felt uncomfortable to tape record, notes were taken. Transcriptions of the various interviews were made to allow for theme identification.

### Data analysis

The interviews were conducted in English as the official language. The audio interviews were independently transcribed verbatim in English by the first two authors. Thereafter, transcriptions were checked for consistency by the same authors.

Because of the qualitative nature of the study, independent inductive thematic analysis was used to analyse the data by the first two authors. This allowed for the transcribed data to be organised into meaningful categories and themes. This allowed for themes to be extracted from the transcriptions. After extraction, co-authors cross-checked the themes to double-check and ensure they correlate with the coded data. The review of themes against the coded data was conducted until data saturation was reached, and no additional themes could be extracted.

### Ethical considerations

Ethical clearance to conduct this study was obtained from the University of Namibia, Department of Psychology & Social Work Research Ethical Clearance Committee (No. PS-SoAHS-FHSVM-2022/4). Voluntary informed consent was obtained verbally and dated accordingly with participants’ initials before the interview processes began. Given the nature of the sample, no written consent could be obtained as participants needed the consent form in braille format, which was not possible to acquire at the time of data collection. No identifiable credentials were used. The data collected were stored in a password-protected computer with access available only to the researchers. For the participants who consented to being recorded, the audio files were deleted as soon as the transcribing was conducted to uphold confidentiality and anonymity.

## Results

As shown in Table 1, nine participants formed the sample of the study. The sample comprised two male participants and seven female participants (all with visual impairment). The participants were all in possession of a degree as their highest qualification. Although qualified, three participants were unemployed and searching for employment opportunities at the time of the study.

**TABLE 1:** Demographic characteristics of study participants.

Demographic	Grouping	*n*	%
Gender	Male	2	22
	Female	7	78
Age group (years)	20–30	2	22.2
	31–40	4	44.4
	41–50	2	22.2
	51–60	1	11.1
Highest education qualification	Certificate	2	22.2
	Diploma	3	33.3
	Degree	4	44.4
Interpreter during interview	Yes	0	0
	No	9	100

Through inductive thematic analysis, *six themes* pertaining to the employment challenges of securing and maintaining employment faced by persons with visual impairments are presented.

### Experiences on seeking employment

#### Theme 1: Mixed experiences of excitement, rejections and discouragement

Participants reported mixed experiences. Some participants’ experiences were positive as they felt they did not have a different experience as compared to their peers without disabilities. They understood that there would be some rejections, but that was to be expected in the employability journey. Some of them were also fortunate enough to have had employment waiting for them by the time of completing tertiary education, while others were employed at institutions for the visually impaired, so their transitions into working life was smoother than for others.

In addition, some participants did not view their disability as an issue. Instead, they believed that what they were able to bring to the table was a lot more important. They believed that their impairment would not drag them down and be the basis on which they would be judged, but rather that their abilities should have been the bases thereof. These participants believed that it was what you brought to the table, or what you had to offer concerning your skills and knowledge qualifications that carried greater weight, despite the disability you may have.

Participant 1 explained:

‘[*P*]eople do not necessarily see you they only see your resume, from there it is not about looks, but what you can bring to the table; and that definitely helps.’ (Participant 1, female, 41–50 years)

Other participants had negative experiences, often marked by frustrations and discouragement. Participant 9 voiced her experience in searching for employment:

‘Tiring, and extensive. At that time, every now and then, you see a position being advertised; you would have to make plenty of copies, and if you couldn’t do it yourself, you would have to get an assistant to go with you to wherever the place is. In most cases, what is weird is you would see your colleagues without disabilities being called up for interviews, and you were never called up for an interview.’ (Participant 9, female, 31–40 years)

Participant 4 added:

‘As I indicated, people won’t get back to you. There just won’t be any response, even if you had to drop you[*r*] applications or apply for as many position as you want, you just don’t get invited for an interview. It’s like you’re trying to go up a ladder, and in the 1st place no one even allows you to get to the ladder. So you don’t even know whether you can climb, or cannot climb.’ (Participant 4, male, 31–40 years)

It was evident that majority of participants encountered challenges when seeking employment. The job application process was different or more difficult in their opinion. They felt shut down before they even had a chance to portray their competency or skills. Participant 2 mentioned:

‘[*M*]y dear, the man that was hiring accepted my application and even sounded positive for the interview. Once I came in for the interview, the mood changed. He even asked me how will I deal with the environment, like if I needed to use the bathroom, who would help me. You know, those types of questions just demoralise you and show you what people think of you.’ (Participant 2, female, 31–40 years)

Participants stated that it took quite a while for employers to recognise their potential because of their disability. This resulted in them having to work harder to prove themselves, thus further reinforcing the pressure they exerted on themselves. Participant 5 admitted that:

‘[*T*]hey think of you like this poor thing that cannot do things. They don’t even allow you to showcase your skills and stuff. They feel like you are just going to be [*a*] burden and need help all the time.’ (Participant 5, female, 20–30 years)

This pressure, according to some participants, resulted in stressful experiences. Some participants worked in careers geared towards advancing the visually impaired, and the fact that they were disabled gave them an advantage in terms of preferential treatment.

Participants placed great emphasis on relying on their abilities when searching for a job. They believed that ‘if you know who you are and what you brought to the table’, you could achieve anything.

Participant 6 explained:

‘[*I*]f you know what you want, I do not think that your disability will be a barrier from where you want to go or where you need to go. It is about where you want to go; you cannot let the impairment, or the disability hold you back.’ (Participant 6, female, 31–40 years old)

However, some participants experienced some challenges. They questioned their competency and their connectedness to others as they were under the impression that people without disabilities achieved their goals a lot quicker than those with disabilities.

Participant 7 stated:

‘I would always feel like people around me could do whatever they were doing better than I did. I could not always keep up, and I needed to work so much harder. Obviously, that affected my independence because I always felt like I was not competent.’ (Participant 7, female, 31–40 years)

The experiences affected their emotions to a point where they were experiencing more negative emotions which influenced their life satisfaction. Negative emotions ranged from self-doubt and a lack of belief in their abilities, and this further contributed to experiences of more negative emotions.

Participant 2 was quite vocal about her difficulty in dealing with her emotions with regard to looking for employment:

‘The struggle to find a job was demoralising. It makes you want to give up and start wondering why you are the one that was born with visual impairment. The constant rejection starts to feel like there is something wrong with you, you know. To be honest, it is not easy staying positive through it all, but ya.’ (Participant 2, female, 31–40 years)

Participant 8 added:

‘[*G*]rowing up, you were made fun of, so emotionally, it was hard because I always had this subconscious thing of, “[*T*]hese people are looking down on me”, even if it may not have been the case. Emotionally, I had to try and control what was perceived and what was done, and finding this balance was hard.’ (Participant 8, female, 20–30 years)

#### Theme 2: Lack of assistance from companies and organisations

Participants noted how discouraging it was to prepare your CV, only to come to the company and there is no one willing to assist you to submit your application for employment:

‘This is because you don’t know where to go to hand in applications or go to enquire about the progress of the application processing. Some of the people are just not helpful sometimes you know.’ (Participant 2, female, 31–40 years)

Participant 5 narrated that:

‘I can familiarize myself quite well with places I know but sometimes you need assistance, but you don’t always get it. Sometimes maybe there are notices at the entrance about where to submit this and that, but you can’t see so you can’t also read.’ (Participant 5, female, 20–30 years)

#### Theme 3: The role of positive and supportive relationships

Few of the participants felt supported by their community and in turn experienced a sense of belonging while attempting to secure employment.

Participant 1 noted:

‘I was very lucky because I started in a special school already. So, I didn’t feel awkward, and especially now because I am teaching the visually impaired kids. It is not a challenge because I knew how to handle things because I also came from the visually impaired school. I also went through that process.’ (Participant 1, female, 41–50 years)

Participant 7 added:

‘I had a great support system.’ (Participant 7, female, 31–40 years)

Participant 2 felt that her community looked up to her as a good role model, but in the same vein, she identified with her communities’ role models. Her integration and acceptance into her different communities was based on mutual admiration and mutual respect.

Participant 2 expressed:

‘I had a small group of friends, and having them around makes it easier than keeping a crowd, if that makes sense. And being accepted by these five friends, we were pretty close, and some of us are even still close; it has been 25+ years. You just realise that being accepted by a smaller group of people brings more joy to your heart than being accepted by a crowd.’ (Participant 2, female, 31–40 years)

Some participants experienced more positive emotions during the journey of searching for employment. This was because some of them experienced positive support that encouraged them to be satisfied with the life they had. Participant 5 and Participant 1 expressed that their communities helped enhance the positivity they experienced during that time.

#### Theme 4: Mental fortitude and spirituality

This theme focused on participants’ ability to cope with the various experiences that come with searching for employment.

Participant 9 stated:

‘[*P*]sychologically, it’s just the same. I believe in myself. The first thing that made me to believe is that I went through the same thing, classes and studies with others. We failed together, and we passed together. I didn’t fail because of my disability but because I did not study, or understand, or enjoy the subject, just like everyone else. That’s why I never doubted myself or seen myself as less of a person. I view myself as the best of what I do; that’s what makes me wake up in the morning.’ (Participant 9, female, 31–40 years)

Most participants relied on their religion or spirituality to guide them through tough times or be grateful for the good times.

Participant 2 noted:

‘[*T*]his is my passion and fate; God wouldn’t have led me here.’ (Participant 2, female, 31–40 years)

Other participants truly struggled to gain employment, but Participant 3 knew that:

‘[*M*]y religion is strong my dear; God gives his hardest battles to his strongest soldiers.’ (Participant 3, female, 51–60 years)

Some participants were working in the field of visually impaired advancement and the fact that they themselves fell into the community gave them an advantage in terms of preferential treatments. In that regard, their disability directly impacted their experience in searching for employment. Participant 1 and 5 both work at institutions for persons with visual impairment and have admitted that they knew the easiest place of employment to look for was in those environments as their input would be appreciated more. Participant 3 was able to get into her current job because she was able to get into the specific tertiary institution because of initiatives such as affirmative action.

Participant 5:

‘I applied to the Federation for the Visually Impaired so securing employment as a person with disability was fairly easy because there was no discrimination and they understood the need for our community to have a job.’ (Participant 5, female, 20–30 years)

A sense of happiness and contentment with where they were in their lives was highlighted in some of the interview discussions. This was evident with participants 3, 4, 5, 6, 7 and 8.

### Challenges in maintaining employment

Participants 2, 4 and 7 had not secured employment and therefore made minimal contributions in this section.

#### Theme 5: Challenges of isolation and frustration

All other participants, except for participants 2, 4, 7, indicated that, at times, one would think you have made progress but then you move two steps back because of one or other issue, that is sometimes beyond your control. This sometimes pushes individuals to quit their work because of a lack of growth prospects.

The participants experienced a sense of unease regarding their perceived competency. This was a result of the judgement that they received from their colleagues. They expressed that they felt incompetent because they could not always complete tasks in the same manner or timeframe as their peers. They realised that this experience would have been better had they experienced social acceptance.

Participant 2 stated:

‘I always felt a little judged by my colleagues because of my impairment. I sometimes felt like they thought I was putting on a show when I was struggling and that they were always looking down on me and judging me for my disability. They would simply think that I was just looking for excuses not to perform tasks at work, but I do believe that I proved myself, regardless of my impairment.’ (Participant 2, female, 31–40 years)

#### Theme 6: Challenges of adaptation and flourishing

The participants mentioned that another factor that influenced maintaining employment was the need to adapt. According to the participants, there was an urgent need for them to be prepared for instances where they knew someone without disabilities would not accommodate them. Hence, adaptation needed to become their second nature to safeguard their well-being.

Participant 8 mentioned:

‘[*W*]e work with laptops, so that has a big effect on productivity, which is hard because you can’t even do what others can because of factors such as light, color, where you sit in the office, what technology is available to you, so adjusting was difficult. You need a lot of adaptation and on the spot thinking and comprising because I had to start thinking, “[*W*]hat can I do if something doesn’t accommodate me?”.’ (Participant 8, female, 20–30 years)

Participant 7 stated:

‘Awareness was minimum in the workplace, when you hear people speak about people with disabilities, it would be a shock. They ask questions like how are they working, how are they performing their work if they cannot see. I remember someone asking, “[W]hy me? Why did you need to come to my division?” They thought I would waste their time by coming in, sitting around and wasting their time. Which is actually the wrong perception.’ (Participant 7, female, 31–40 years)

It was noted, from participants’ narratives that in some workplaces, that they would also come across situations where people claim that devices or equipment especially for accessibility used by people with disabilities are very expensive. So they would rather avoid the cost.

Despite the challenges documented earlier in the text, for other participants who had managed to maintain employment, a recurrent theme of inclusion emerged strongly during the data-collection process. The feeling of being part of a work community and feeling like they could add value in improving and changing that specific community meant a lot to the participants.

#### Theme 7: Positive experiences of inclusion

Participant 8 was very vocal noting that she had to fight against negativity to acquire the job; but once she was a part of the institution, she felt part of the work community. She noted:

‘I felt isolated in tertiary education and maybe that’s because people were young, and did not really understand. But in the work environment, everyone does their part and that makes everything run smoother. I no longer felt isolated.’ (Participant 8, female, 20–30 years)

Like the aforementioned, even with the difficulties they faced in trying to secure employment, participants’ emotional well-being was significantly improved because they were able to acquire employment. Participant 1 and Participant 6 repeatedly expressed feelings of gratitude that improved their positive emotionality as well as their job and life satisfaction.

Participant 6 continued on to say:

‘[*M*]y dear, how can one not be grateful for the opportunity to work in the field you always dreamed to be a part of? I am just so happy; it’s like I cannot even explain it properly.’ (Participant 6, female, 31–40 years)

Participant 1 spoke of the day she graduated and what that meant to her because she already knew she had a job waiting for her, but the graduation meant that she had finally made it through.

A recurrent pattern in this section of the interviews was that their emotional well-being improved with the ability to maintain employment. Their understanding of the struggles that come with maintaining employment for those with disabilities left them generally feeling more optimistic and positive about their jobs, and therefore their lives.

Participant 6 mentioned:

‘I think with the well-being, [*it*] starts with, “[*C*]an you maintain what you said you can do?” because if you can maintain what you promised in the interview, obviously no one will look at you or treat you badly or think that you were given the position because you are visually impaired, like you are a charity case, because if you can provide the goods, no one tends to ask questions because you keep them happy.’ (Participant 6, female, 31–40 years)

In fact, their ability to acquire and maintain employment increased their sense of competency and autonomy. Participant 3 experienced high levels of autonomy and mastery because:

‘[*T*]he work life is much better than my tertiary experience. I get to do my part and do it well, and I don’t feel like a burden anymore. In fact, I feel like an asset.’ (Participant 3, female, 51–60 years)

Participant 2 has not been able to find paid employment and occasionally volunteers her counselling skills at an institution or her church. Her frustration stemmed from when she would volunteer at institutions because:

‘I would volunteer at these places and they would really be impressed with my skills and how I handle some cases. But the problem would always be when we get to the point of permanent employment, there was always an issue. There was always a waiting list of who can be hired and I would be put on that list but there are a lot of people and you cannot volunteer forever because we also need to support ourselves. These waiting periods don’t help us.’ (Participant 2, female, 20–30 years)

## Discussion

The various themes uncovered reveal negative and positive employment experiences of persons with visual impairment. It is an undeniable fact that the standard of living for persons with disabilities improves significantly with securing and maintaining employment (Cimarolli & Boerner 2005).

The findings of the study revealed the challenges involved in the job applications for persons with visual impairment and the lack of reasonable accommodations adopted by workplaces in efforts to facilitate this process. In view of this, it is crucial that the Human Resources Management offices of all workplaces devise strategies to support job applicants with visual impairment in submitting their job applications.

Based on the themes, discrimination and unfairness were emphasised as being prevalent. The experiences of discrimination were noted in some workplaces. This justifies the adoption of the social model on disability, alluding to the fact that participants experience unwarranted isolation and exclusion as a result of systems and structures that exist within society and in workplaces. Amin et al. (2021) indicated that visually impaired students face the challenges of peer-to-peer acceptance at university. The lack of acceptance could be seen as a prevalent issue across various educational contexts. The discrimination that students experience could be argued to stem from a lack of acceptance from fellow human beings in various contexts.

In changing the narrated experiences of persons with visual impairment, the social model of disability argues that the human-made systems and structures that aggravate disadvantages for persons with visual impairment, can be changed.

As such, awareness on types of disabilities and limitations associated with each visual impairment is crucial. Awareness using various educational platforms to reach a wider population can help individuals in society and in workplaces, understand the internal struggles persons with visual impairment endure throughout university and post schooling, in the world of work. It is necessary that individuals and organisations understand the limited abilities of having visual impairment as a key organ of any human being, which becomes inherent to an individual’s optimal functioning in abilities that require the use of vision. This is one way of changing the attitudinal, social and structural barriers embedded in various systems and structures of society, as advocated for by the social model of disability.

The findings reveal that, in some countries, such as in the case of this study, workplaces are not adequately equipped to respond to the needs of employees with visual impairments, and would rather avoid employing such people, because of costs associated with reasonable accommodation. In operationalising the social model and its applicability within the Namibian employment setting, it is crucial that reasonable accommodation measures implemented within organisations are monitored, to ensure the limitations associated with not employing persons with visual impairment are identified and actioned upon at regional and national levels.

The study was able to establish that experiences at the university level for persons with visual impairment create intense mental and emotional pressure for persons with visual impairment in their quest for employment. Although some participants were able to adopt strategies to cope (as shown in the results section), it was in most cases at the expense of their own optimal functioning and wellbeing, which should not be the case. If not addressed effectively, it could have grave potential to cascade into a cycle of emotional and mental turmoil.

As such, for persons with disabilities, inclusion and support are crucial at all levels, in any setting (Manitsa & Doikou 2022; McDonnall & Antonelli 2018). The findings of this study reveal that community support presents a huge support system for persons with visual impairment. The social model emphasises that persons with disabilities come from communities. It is the same communities that influence the perception and understanding of disability. As such, community awareness campaigns tailored towards enhancing an understanding of the challenges experienced by persons with disabilities and the necessary support that could be provided are crucial. This is further corroborated by Cimarolli and Boerner (2005), whose study found that support, especially from families, was linked to positive experiences of well-being for adults with visual impairment. In the context of Namibia, stakeholders can partner at local, regional and national levels in creating support systems for persons with visual disabilities. This could be in the form of assistance with seeking employment and maintaining employment. This can further be enhanced through creating avenues for persons with visual disabilities to belong and contribute to society.

Lund and Cmar (2019) argue that the knowledge and skills acquired at various tertiary institutions play a key role in boosting confidence of graduates with visual impairment to search and maintain employment; however, the lack of recognition for the knowledge and skills acquired, coupled with employer rejection, still had significant impact on their well-being.

This further reiterates the crucial role of advancing the mandate of special and inclusive education to ensure that persons with disabilities are afforded the necessary opportunities to have fundamental education to advance to tertiary education. Schools at the grassroots level and secondary and post-secondary school institutions are urged to continue to find avenues for inclusion and support for students with disabilities. Garcia et al. (2016) argue that securing employment is one way to improve employment outcomes for persons with disabilities. For functional integration in society, the well-being of persons with visual impairment is crucial at every stage of their life (Antonelli et al. 2018b). The findings of the study revealed that the challenges associated with securing employment are vast and have a negative toll on self-acceptance, self-doubt, anxiety and stressful experiences of graduates with visual disabilities, which could potentially impact overall functioning of persons with visual impairment. Individual level interventions (to capacitate persons with visual impairment on ways to safeguard their well-being at different stages in their lives) and organisational level interventions (to harness organisations’ capacity to support well-being of persons with disabilities in their workplaces) should be put in place. To promote optimum integration, Garcia et al. (2016) support the importance of sensitising employers and work environments on the reality of visually impaired persons. According to Steverson and Crudden (2023), positive co-worker relations and supervisor support have been shown to enhance job satisfaction and a sense of belonging for persons with visual impairment. As far as the employment equity or affirmative action policies are concerned, there is need, at regional, national and international level, to develop measures that will strengthen employing organisations’ compliance to the employment and reasonable accommodation measures that come with employing persons with visual impairment. The findings provide an avenue for the government line ministry in charge of disability affairs to engage key stakeholders on crucial issues uncovered in this study.

## Limitations

Despite efforts to have more than 20 participants in the present study, the population of persons with visual impairment in Windhoek proved to be rather limited in number. Future research could strategise on reaching participants spread across the entire country. The study employed a cross-sectional qualitative research method and as such was only able to assess the employment experiences of graduates with visual impairment. Quantitative studies on employment and unemployment and/or related variables should be conducted to enhance understanding of the phenomenon of employment for persons with visual impairment.

## Conclusion

More efforts need to be diverted towards creating an integrative support model for persons with visual impairment, comprising all stakeholders at local, regional and national levels. The reality on the ground is that persons with visual impairment will need a considerable amount of reasonable accommodation, at all levels, to allow for optimal integration into the labour market. The institutions of higher learning and workplaces should provide an environment of inclusion and support for persons with visual impairment to contribute to the development of persons with visual impairment who are resilient and who have a sense of belonging.
